# Comprehensive Review of Aflatoxin and Ochratoxin A Dynamics: Emergence, Toxicological Impact, and Advanced Control Strategies

**DOI:** 10.3390/foods13121920

**Published:** 2024-06-18

**Authors:** Tiago de Melo Nazareth, Elisa Soriano Pérez, Carlos Luz, Giuseppe Meca, Juan Manuel Quiles

**Affiliations:** Laboratory of Food Chemistry and Toxicology, Faculty of Pharmacy, University of Valencia, Av. Vicent Andrés Estellés s/n, 46100 Burjassot, Spain; sorianoperezelisa@gmail.com (E.S.P.); carlos.luz@uv.es (C.L.); giuseppe.meca@uv.es (G.M.); juan.quiles@uv.es (J.M.Q.)

**Keywords:** mycotoxins, outbreaks, mycotoxin prevalence, legislation, essential oils, lactic acid bacteria, climate changes

## Abstract

Filamentous fungi exhibit remarkable adaptability to diverse substrates and can synthesize a plethora of secondary metabolites. These metabolites, produced in response to environmental stimuli, not only confer selective advantages but also encompass potentially deleterious mycotoxins. Mycotoxins, exemplified by those originating from *Alternaria*, *Aspergillus*, *Penicillium*, and *Fusarium* species, represent challenging hazards to both human and animal health, thus warranting stringent regulatory control. Despite regulatory frameworks, mycotoxin contamination remains a pressing global challenge, particularly within cereal-based matrices and their derived by-products, integral components of animal diets. Strategies aimed at mitigating mycotoxin contamination encompass multifaceted approaches, including biological control modalities, detoxification procedures, and innovative interventions like essential oils. However, hurdles persist, underscoring the imperative for innovative interventions. This review elucidated the prevalence, health ramifications, regulatory paradigms, and evolving preventive strategies about two prominent mycotoxins, aflatoxins and ochratoxin A. Furthermore, it explored the emergence of new fungal species, and biocontrol methods using lactic acid bacteria and essential mustard oil, emphasizing their efficacy in mitigating fungal spoilage and mycotoxin production. Through an integrative examination of these facets, this review endeavored to furnish a comprehensive understanding of the multifaceted challenges posed by mycotoxin contamination and the emergent strategies poised to ameliorate its impact on food and feed safety.

## 1. Introduction

Filamentous fungi are widely distributed eukaryotic organisms capable of thriving on various substrates by synthesizing diverse secondary metabolites. These metabolites, produced in response to environmental cues, include beneficial substances like antibiotics and industrially valuable compounds such as antioxidants [1]. However, they can also produce harmful mycotoxins, posing significant health risks to humans and animals [2].

Mycotoxins, produced by fungi such as *Alternaria*, *Aspergillus*, *Penicillium*, and *Fusarium*, are potent contaminants in food and animal feeds [3]. Due to their harmful effects, stringent regulations have been implemented to control their presence, driven by both health risks and economic impacts of contaminated batches [4].

Human exposure to mycotoxins occurs directly through contaminated food or indirectly via animal products from mycotoxin-exposed animals [5]. Over 400 mycotoxins have been identified, many of which are heat-stable, persisting even after food processing and posing significant health risks [6].

Cereals, crucial for animal nutrition, are highly susceptible to mycotoxin contamination. To reduce dependence on cereal-based feeds, researchers are exploring alternative options such as crop by-products like barley and almonds, which provide valuable fibers and proteins for livestock, particularly in dairy and beef cattle, swine, and pigs [7].

There is a direct relationship between the initial contamination levels of crops and the subsequent contamination of derived by-products. Additionally, animals exposed to mycotoxins can transfer these toxins to animal-derived food products through a process known as carry-over, where mycotoxins from contaminated feed are found in edible animal tissues or by-products [8]. The processing of cereals results in significant waste, with thirty percent of cereal weight lost, accounting for 13% of global food waste [9]. The brewing industry alone produces vast amounts of waste, including spent grain, yeast, wastewater, skins, shells, and hulls, with 10,000 tons of liquid waste and 137–173 tons of solid waste per 1000 tons of beer [10].

Mycotoxins have diverse chemical and biological properties and can cause acute and long-term health effects in humans and animals, including nephrotoxicity, hepatotoxicity, teratogenicity, carcinogenicity, estrogenicity, and immunosuppression [11]. Despite these risks, only common mycotoxins are regulated globally, with maximum tolerated limits set for foodstuffs, including aflatoxins (AFs), deoxynivalenol (DON), fumonisins (FUMs), ochratoxin A (OTA), patulin, and zearalenone (ZEN). Feedstuff regulations are less stringent, with only a few nations, mainly in Europe and the US, setting limits for mycotoxins like AFs [12].

While reviews often address the dangers of globally regulated mycotoxins like AFs and OTA, they lack in-depth exploration of novel biocontrol methods. This review focuses on AFs and OTA, establishing a foundational understanding and exploring biocontrol approaches using mustard essential oil and lactic acid bacteria (LAB). Aflatoxin B1 (AFB1) and OTA are particularly concerning due to their high prevalence in food products and significant health impacts. Research indicates a strong link between these mycotoxins and various health issues [13].

**Figure 1:**
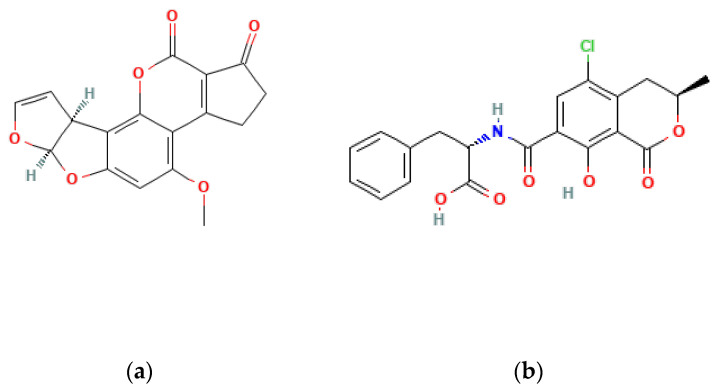
Chemical structure of AFB1 (**a**) and OTA (**b**).

AFB1 has become a major focus of scientific inquiry and strict regulatory measures due to its severe health and economic impacts. This prominence stems from their potent carcinogenic properties and the associated economic losses (Figure 1) [14]. Classified as a Group 1 carcinogen by the International Agency for Research on Cancer (IARC), it is considered one of the most potent natural carcinogens known [15]. Exposure to AFB1 is linked to liver cancer and various other liver diseases in humans, including jaundice, necrosis, cirrhosis, and hepatitis. Additionally, it can cause non-hepatic health problems like immunosuppression and impaired growth [16]. It is important to remember that Group 1 carcinogens are confirmed to have harmful effects on both humans and animals.

OTA is a major concern due to its negative health effects (Figure 1). This mycotoxin can damage the kidneys (nephrotoxic), suppress the immune system (immunosuppressive), and potentially cause cancer (carcinogenic). The International Agency for Research on Cancer (IARC) has classified OTA as a Group 2B carcinogen, indicating that it is possibly carcinogenic to humans. For reference, IARC classifies mycotoxins based on the strength of evidence linking them to cancer: Group 1 (confirmed), Group 2A (probable), and Group 2B (possible) [17]. The most common and harmful mycotoxins, as well as the level of evidence for carcinogenicity, are listed in Table 1 according to the IARC.

The global prevalence of AFB1 and OTA requires the development of effective control strategies to prevent their presence in food or minimize their harmful effects. Traditional methods have relied on fungicides and pesticides to control toxigenic fungi. However, concerns regarding their inherent toxicity have led to stricter regulations on their use [18]. Biological control using specific microorganisms like LAB and certain fungal strains has emerged as a promising alternative [19]. Detoxification methods are also employed to mitigate the effects of existing toxins [20]. Despite these efforts, challenges remain. Studies continue to report high levels of mycotoxin contamination, particularly in regions conducive to fungal growth [21,22]. This highlights the need for exploring additional solutions.

In recent years, there has been growing interest in natural alternatives like essential oils (EOs) derived from plants and spices. Research suggests that EOs possess antifungal properties and can inhibit the growth of harmful pathogens in food [23,24,25]. Essential mustard oil is one such example with potential application.

This review will delve into the limitations imposed by AFs and OTs, including their presence in raw materials and their byproducts, the associated health risks for humans and animals, recent outbreaks, and current legislation regulating their levels in food and feed. Additionally, the review will explore traditional methods of mycotoxin prevention, biopreservation as an alternative to biocides, and the antifungal efficacy of EOs with a particular focus on the potential of essential mustard oil.

## 2. AFs and Fungal Producers

AFs contamination in crops is a complex process triggered by fungal invasion and toxin production within the affected crops. Several factors influence this process, including environmental conditions, the type of crop, and the specific fungal strain involved. Not all *Aspergillus* species are capable of aflatoxin synthesis, and their ability to infect crops varies. Therefore, the extent of aflatoxin contamination depends heavily on the predominant fungal strain in a particular region [26].

Extensive research has identified fungal species with aflatoxin-producing capabilities. While modern molecular techniques have improved our understanding, significant gaps in knowledge remain regarding specific members of the *Aspergillus* genus and their aflatoxin production potential. As shown in Table 2, at least 24 *Aspergillus* species belonging to the *Flavi*, *Nidulantes*, and *Ochraceorosei* sections are capable of producing AFs [27].

*A. flavus*, *A. nomius*, and *A. parasiticus* are the primary responsible for aflatoxin production, with other species like *A. astellatus* also contributing [28]. This toxin biosynthesis is a complex process involving at least 27 enzymes, with genetic analysis revealing similarities among aflatoxin-producing fungi [29]. Furthermore, around 30 genes are known to be crucial for aflatoxin synthesis [30].

Fungi belonging to the *Aspergillus* genus thrive in warm and humid environments, with optimal growth occurring at temperatures around 25 °C and a wide pH tolerance range (2–11.2). Additionally, they require a water activity near 0.85 for optimal growth [31]. Cereals, fruits, nuts, and seeds are particularly susceptible to fungal infection during storage, making them high-risk products for aflatoxin contamination.

**Table 2:** Fungal species that were previously identified as aflatoxin producers.

Species	Aflatoxin	Provenance	Reference
*Aspergillus flavus*	B1, B2, G1, G1	Nuts, cereals, and several other commodities	[32,33]
*A. parasiticus*	B1, B2, G1, G2	Peanut, maize	[34]
*A. bombycis*	B1, B2		[33]
*A. pseudotamarii*	B1, B2	Cereals and soil	[34,35]
*A. nomius*	B1, B2, G1, G2	Wheat, Brazil nuts, and other substrates	[36]
*A. toxicarius*	B1, B2, G1, G2	Chestnuts	[37]
*A. parvisclerotigenus*	B1, B2, G1, G2	Maize	[38]
*A. columnaris*	B1, B2, G1, G2	Maize	[33,39]
*A. zhaoqingensis*	B1, B2	Cereals and soil	[35,40]
*A. novoparasiticus*	B1, B2, G1, G1	Maize	[41,42]
*A. mottae*	B1, B2, G1, G2	Cereals, Brazil nuts, almonds, figs, pistachio nuts	[43]
*A. sergii*	B1, B2, G1, G2	Cereals, oilseeds	[15]
*A. pseudocaelatus*	B1, B2, G1, G2	Maize	[41]
*A. transmontanensis*	B1, B2, G1, G2	Cereals	[15]
*A. luteovirescens*	B1, B2, G1, G2	Cereals	[34]
*A. minisclerotigenes*	B1, B2, G1, G2	Peanut	[44]
*A. arachidicola*	B1, B2, G1, G2	Maize, Arachis glabrata	[41]
*A. austwickii*	B1, B2, G1, G2,	Cereals	[44]
*A. aflatoxiformans*	B1, B2, G1, G2	Cereals	[34,44]
*A. pipericola*	B1, B2, G1, G2	Cereals	[15]
*A. cerealis*	B1, B2, G1, G2	Cereals	[15]
*A. Togoensis*	B1, B2	cereals	[15]
*A. venezuelensis*	B1, B2	Cereals	[15]
*A. astellatus*	B1, B2	Cereals and other substrates	[15,33]
*A. miraensis*	B1	Cereals	[15]
*A. olivicola*	B1	Cereals	[15]
*A. ochraceoroseus*	B1, B2	Cereals	[33,45];
*A. rambellii*	B1	Cereals	[45]

**Figure 2:**
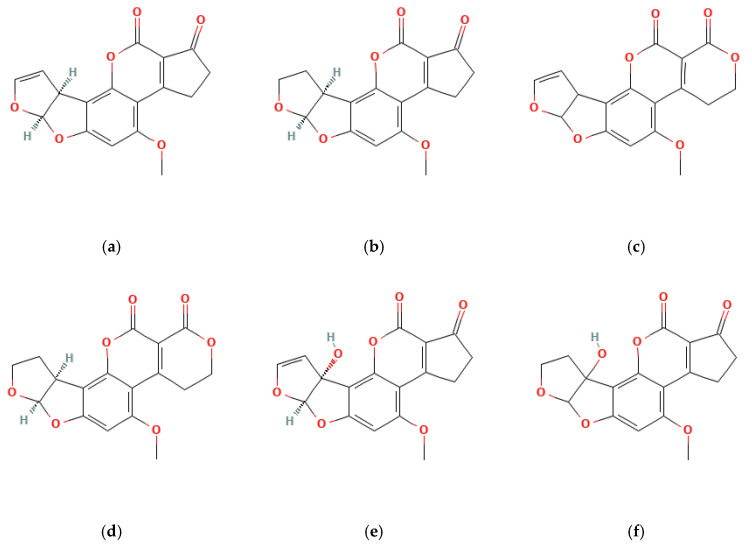
Chemical structure of the five most toxic aflatoxins. AFB1 (**a**); AFB2 (**b**); AFG1 (**c**); AFG2 (**d**); AFM1 (**e**); AFM2 (**f**).

AFB1, aflatoxin B2 (AFB2), aflatoxin G1 (AFG1), and aflatoxin G2 (AFG2) are the primary aflatoxin contaminants, distinguished by their fluorescence under ultraviolet light and chromatographic properties (Figure 2) [46]. These belong to the furanocoumarin group and represent a fraction of the over dozen AFs identified. Many of these toxins are formed through the breakdown of four main fungal-derived compounds found in food by the liver [47]. For instance, AFB1 is metabolized by the liver’s cytochrome P450 enzymes into various metabolites, with aflatoxin M1 (AFM1) being the most significant. AFM1 is produced through the hydroxylation of a specific carbon atom within the molecule.

A key mechanism underlying their genotoxicity involves the formation of a reactive AFB1 epoxide through the action of cytochrome P450 enzymes, which will be discussed in a later section [48].

## 3. OTs and Fungal Producers

OTA was first discovered in South Africa from a culture of *A. ochraceus* growing on maize meal [49]. Since then, other fungal species have been identified as OTA producers, including *A. niger* and *A. carbonarius*, which are commonly found on grapes and other fruits exposed to sunlight and high temperatures [50]. Additionally, several *Aspergillus* species, like *A. tubingensis*, *A. westerdijkiae*, and *A. steynii*, and *Penicillium* species, like *P. verrucosum*, and *P. nordicum*, have also been linked to OTA production [51]. Table 3 summarizes the currently known *Aspergillus* and *Penicillium* species capable of producing OTA in food.

**Figure 3:**
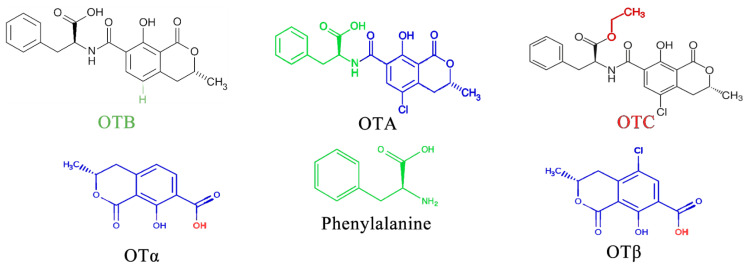
Types of OTs and OTA composition.

OTA is the most prevalent and toxic among OTs [52]. Research has identified over 20 OTA metabolites produced by *Aspergillus* and *Penicillium* molds, including ochratoxin B (OTB) and ochratoxin C (OTC). These mycotoxins belong to the pentaketide family and share a similar structure—an isocoumarin molecule (sometimes chlorinated) linked by an amide bond to an L-phenylalanine unit. While structurally similar, these OTs have slight variations. For example, OTB lacks the chlorine atom present in OTA, and OTC has a different radical group attached (phenylalanyl and ethyl ester) [53]. These variations significantly impact their toxicity, with OTA being the most potent and frequently encountered [52] (Figure 3).

Similar to aflatoxin-producing fungi, OTA-producing species are found globally in various food products. Environmental factors like temperature and humidity significantly influence OTA production. In colder climates, *P. verrucosum* and *P. nordicum* are the primary species, while *A. ochraceus* dominates in tropical and subtropical regions [54,55]. It is important to note that these fungi can often produce multiple toxins simultaneously. The co-occurrence of OTA with other mycotoxins like OTB and OTC raises concerns about potential synergistic toxic effects [56].

OTA production is highest at a water activity (aw) of 0.98, regardless of temperature, and further increases within the optimal temperature range of 25–30 °C [57]. OTA is an organic acid with a pKa of 7.1 and a molecular weight of approximately 404 g/mol. It appears colorless to white in crystalline form and exhibits fluorescence under UV light—green in acidic and blue in alkaline conditions. OTA shows good solubility in various organic solvents like alcohols, ketones, and chloroform under acidic and neutral conditions. In alkaline environments, it dissolves in aqueous sodium bicarbonate solution and other alkaline solutions [58,59]. A notable characteristic of OTA is its high stability under acidic and high-temperature conditions, making it difficult to eliminate once food is contaminated. Even standard cooking processes and heating under various moisture conditions up to 200 °C have minimal impact on its structure or decomposition [60].

**Table 3:** Species of fungi reported as OTA producers in Genus *Aspergillus* and *Penicillium*.

Species	Provenance	Reference
*A. affinis*	Decomposing leaves	[61]
*A. cretensis*	Soil	[61]
*A. elegans*	Bread, sponge	[61]
*A. flocculosus*	Grapes	[61]
*A. melleus*	Cereal	[61]
*A. muricatus*	Peanuts	[61]
*A. ochraceopetaliformis*	Sponge	[61]
*A. ostianus*	Pulses	[61]
*A. ochraceus*	Soya beans, nuts, red pepper, cereals, green coffee beans	[62]
*A. pseudoelegans*	Soil	[61]
*A. pulvericola*	Indoor house dust	[61]
*A. roseoglobulosus*	Decaying leaves	[61]
*A. sclerotiorum*	Apple	[63]
*A. steynii*	Barley, coffee, grapes	[62]
*A. subramanianii*	Shelled nuts	[61]
*A. sulphureus*	Soil	[63]
*A. westerdijkiae*	Decomposing leaves	[62]
*A. alliaceus*		[63]
*A. albertensis*		[63]
*P. verrucosum*	Cereals	[54]
*P. nordicum*	Cereals and meat products	[54]
*P. thymicola*	Canadian cheddar cheese	[61]
*P. radicicola*	Carrots and potatoes	[54]
*P. viridicatum*		[54]
*A. carbonarius*	Grapes, red pepper, coffee beans	[64]
*A. awamori*		[64]
*A. awamori var. fumeus*		[64]
*A. foetidus*	Grapes	[64]
*A. lacticoffeatus*	Coffee beans	[63]
*A. niger group*		[64]
*A. niger*	Grapes, raisins, maize, coffee, beer	[64]
*A. japonicus*		[64]
*A. sclerotioniger*	Coffee	[62]
*A. tubingensis*	Grapes	[62]
*A. welwitschiae*	Grapes, raisins, pistachio, walnuts	[61]
*P. expansum*	Pomaceous fruits and nuts	[54]

Studies using radiolabeled precursors have shed light on the complex biosynthesis of OTA. Experiments with *A. ochraceus* cultures employing 1-^14^C-phenylalanine demonstrated that L-phenylalanine serves as the precursor for the toxin’s phenylalanine moiety [63]. Similarly, the isocoumarin portion of OTA primarily originates from acetate condensation, as evidenced by the incorporation of 2-^14^C-sodium acetate.

Further confirmation regarding the precursors involved comes from precursor-feeding experiments with *P. verrucosum* cultures. The addition of both 2-^14^C-sodium acetate and 2-^14^C-malonic acid into the liquid culture medium resulted in their incorporation into OTA biosynthesis. While the entire OTA molecule could be labeled using 2-^14^C-sodium acetate, only the isocoumarin moiety exhibited exclusive labeling with 2-^14^C-malonic acid. These findings suggest that acetate serves as a precursor for both the phenylalanine and isocoumarin moieties, while malonic acid is specifically involved in isocoumarin biosynthesis [63].

## 4. Prevalence of Mycotoxins in Food and Feed

Food quality and safety are influenced by intrinsic factors (e.g., chemical composition, water activity, pH) and extrinsic factors (e.g., environmental conditions during storage). Molds thrive best at room temperature with sufficient oxygen but can also grow under refrigeration [65]. Warm and humid climates, particularly in developing countries with inadequate grain storage, significantly increase mycotoxin contamination risks [66]. The FAO/WHO estimates that roughly 25% of global grain production exceeds permissible mycotoxin levels, leading to an annual loss of about 1 billion tons of grains and flour. Studies in Europe and the US highlight widespread mycotoxin presence, with significant economic impacts [67,68].

Mycotoxin contamination can occur at various stages of the food chain, including pre-harvest, harvest, drying, and storage. Inadequate agricultural practices, poor handling, and improper storage conditions contribute to fungal growth and mycotoxin contamination [69]. Once produced, mycotoxins permeate the entire fungal colony, including hyphae, mycelium, spores, and the surrounding substrate. Consumption of contaminated food is the primary exposure route, though inhalation and skin contact also pose risks [70].

Human and animal consumers are often exposed to multiple mycotoxins simultaneously due to several factors: multiple fungal species producing the same mycotoxin, a single fungus producing various mycotoxins concurrently, and food/feed mixtures containing different toxins [71]. The global demand for cereals like barley, corn, soybeans, and wheat is increasing, making food and feed safety paramount [72]. Mycotoxins represent a growing threat in this context [73].

A comprehensive survey by Khodaei et al. [74] highlighted the prevalence of mycotoxins in cereals like wheat, corn, and rice. AFB1 levels often exceeded EU limits. Climate change is contributing to shifts in fungal populations and mycotoxin distributions, necessitating practical control and management strategies to safeguard crop integrity and consumer health.

Global data indicate significant mycotoxin presence in food. AFs were detected in 55% of raw cereal grains, with the highest level reaching 1642 μg/kg. OTA was found in 29% of samples, with a maximum level of 1164 μg/kg. FUMs, DON, and ZEN were also prevalent. Recent surveys by Streit et al. and Kovalsky et al. reported mycotoxins in approximately 72% and 79% of feed samples, respectively, higher than the FAO’s 25% estimate [75].

A ten-year global survey of nearly 75,000 feed and feed ingredient samples from 100 countries (2008–2017) revealed that 88% contained at least one mycotoxin, with regional variations influenced by climatic factors [76]. Another study (2004–2011) on agricultural commodities found mycotoxins in 72% of over 19,000 samples, with a concerning rise in aflatoxin contamination [77]. Furthermore, surveys on food grains suggest that the incidence of mycotoxins can range between 60% and 80%, depending on various factors such as the specific mycotoxin of concern, the type of food, the analytical methods employed, and the equipment’s detection limits [68,75].

In the analysis conducted by Khodaei et al. [74], a comprehensive survey scrutinized the most recent investigations pertaining to the prevalence of mycotoxins in various cereals, namely wheat, corn, rice, oats, barley, rye, and sorghum, spanning from 2018 to 2020. The findings underscore a notable emphasis on studies concerning corn, wheat, and rice over the past three years. Particularly concerning are the results indicating a significant hazard posed by AFB1 in these cereals, with levels surpassing the established EU limits in a substantial portion of the examined studies. The seriousness of this issue is exacerbated by the ongoing effects of climate change, which contribute to shifts in fungal populations and mycotoxin distributions across different geographical regions and crops. Consequently, urgent attention is warranted towards the development and implementation of practical control and management strategies to safeguard crop integrity and consumer health.

Recent comprehensive surveys indicate that mycotoxin contamination might be even more widespread than previously estimated. Studies by Streit et al. [78] and Kovalsky et al. [79] reported mycotoxin presence in approximately 72% and 79% of analyzed feed samples, respectively. These figures are considerably higher than the 25% reported by the FAO, highlighting the need for further global investigation [74].

The increasing prevalence of mycotoxin contamination suggests a link to global climate change rather than isolated climatic events [80]. Even after industrial food processing, mycotoxins can persist due to their stability at high temperatures [81]. Research using in vivo and in vitro models suggests potential interactions between different mycotoxins, which can be antagonistic, additive, or synergistic depending on the specific mycotoxins involved [71].

### 4.1. Aflatoxin Occurrence

AFs, particularly AFB1, are toxic and carcinogenic contaminants found in various food and feed products [82]. These toxins are primarily associated with fungal growth during pre- and post-harvest stages in crops like peanuts, maize, wheat, rice, barley, and spices, as well as in commercial goods such as peanut butter, cooking oil, and cosmetics. Table 4 summarizes recent studies highlighting the prevalence of AFB1 contamination in diverse commodities over the past decade.

Several factors influence a crop’s susceptibility to fungal infection and subsequent toxin production. These factors can be broadly categorized into environmental and intrinsic characteristics:

1. Environmental factors: Moisture content, temperature, and storage conditions significantly impact fungal growth and toxin production [83,84].
2. Intrinsic crop characteristics: A crop’s inherent properties like nutritional content, pH, and genetic makeup also play a crucial role. Cultivating crops adapted to their specific environmental conditions can enhance their resistance to fungal spoilage [85].

It is important to note that environmental and intrinsic factors often interact. For instance, while the effects of environmental conditions on food susceptibility are well documented, these factors work synergistically with a plant’s inherent characteristics to influence fungal invasion and toxin production [86].

A crop’s nutritional composition can significantly impact its susceptibility to aflatoxin contamination. Studies have shown that an increase in soluble sugar content within the crop can lead to a substantial rise in AFB1 production by fungi [87]. Similarly, the presence of maize oil has also been linked to increased aflatoxin levels.

Water activity (aw) is another critical factor [88,89,90]. Maintaining grain water activity at or below 0.9 can significantly restrict AFB1 contamination post-harvest [91,92]. However, it is crucial to remember that factors like water activity, temperature, and nutritional content are interrelated and collectively influence aflatoxin synthesis [93].

In a study conducted by Keutchatang et al. [94], AFs and OTA were assessed through the enzyme-linked immunosorbent assay (ELISA), while dietary exposure was appraised using a deterministic approach. Out of the 900 households surveyed, chicken and eggs emerged as the most frequently consumed items, with daily consumption rates reported at 41% and 69%, respectively. However, the study revealed a concerning lack of awareness among the population regarding mycotoxins and their associated health risks, with only 18% displaying any significant knowledge on the subject. Analysis of poultry tissues indicated that mean concentrations of AFs, AFB1, and OTA fell below the established regulatory thresholds in feeds, which are set at 20 μg/kg for AFs, 10 μg/kg for AFB1, and 5 μg/kg for OTA. However, these toxins were detected in chicken muscle and eggs at average concentrations of 1800 and 966.7 ƞg/kg for AFs and 1400 and 1933.3 ƞg/kg for OTA, respectively. Despite these concentrations being below regulatory limits, the estimated daily intakes of AFB1 and OTA through poultry products tended to remain below the respective thresholds of 1 and 100 ƞg/kg bw/day. Margin of exposure (MOE) calculations across different age groups within the population revealed that public health concerns associated with mycotoxin presence in poultry products should not be disregarded [94].

Reducing and effectively managing aflatoxin contamination in food is essential. The degree of contamination directly correlates with the level of human exposure. Therefore, identifying and quantifying mycotoxins in food and animal feed is a crucial aspect of ensuring global food safety, as these contaminants pose chronic health risks.

**Table 4:** Reports of AFs occurrence in food commodities over the last ten years.

Food	Country	Percentage of Contaminated Samples	Mycotoxin	Concentration (μg/kg)	Method of Detection	Reference
Cereal-based baby foods	Brazil	5% of 60	AFB1	2.8	LC-MS/MS	[95]
Cereals	Croatia	3.4% of 89	AFB1	9.0	HPLC-MS/MS	[96]
Dried Fruits	Iran	56.8% of 88	AFB1	0.3 to 8.4	HPLC-FLD	[97]
Dry Fruits	Pakistan	86.7% of 52	AFTotal	0.0242	HPLC-FLD	[98]
Durum Wheat	Tunisia	54.44% of 90	AFB2	0.12–0.58	HPLC-FLD	[99]
Maize flour	Iran	80.0% of 10	AFB1	<LOQ–1060	UHPLC-MS/MS	[100]
Maize	Brazil	25.7 and 7.4% of 148	AFB1 and AFG1	0.5 to 49.9	HPLC-MS/MS	[101]
Maize	Korea	13.6% of 66	AFB1	0.02 to 0.48	HPLC	[102]
Maize flour	Italy	26.0% of 50	AFB1	0.17 to 3.75	HPLC-FLD	[103]
Maize	Haiti	55.0% of 20	Sum of AFs	185.9 ± 303.9	HPLC-DAD	[104]
Maize	Zimbabwe	23.7% of 338	AFB1	0.57 to 26.6	ELISA	[105]
Maize	Ethiopia	8% of 100	AFB1	26.6	HPLC-MS/MS	[106]
Maize	Serbia	57.2% of 360	AFB1	1.3 to 88.8	HPLC-FLD	[107]
Maize	Uganda	25.8 (256)	AFTotal	0 to 3760	TLC	[108]
Maize	Kenya	100%	Sum of AFs	2.14 to 411	UHPLC-FLD	[109]
Fermented meat products	Croatia	8.3 and 58.3% of 180	AFB1and OTA	<0.05–7.83	HPLC-FLD	[110]
Infant formulae	Mexico	20% of 55	AFM1	0.040 to 0.450	HPLC	[111]
Milk	China	80%	AFM1	0.005 to 0.10	ELISA and HPLC-MS/MS	[112]
Milk	Lebanon	58%	AFM1	0.011 to 7.350	HPLC	[113]
Milk	Nigeria	100% of 25	AFM1	0.081	LC-MS/MS	[114]
Wheat	China	6.2% of 178	AFB1	0.03 to 0.12	HPLC-MS/MS	[115]
Nuts	Zimbabwe	12.5% of 208	AFB1	0.7 to 175.9	HPLC-FLD	[116]
Rice	Ecuador	7.0% of 230	AFB1	4.9 to 47.4	HPLC-FLD	[117]
Rice	Pakistan	18.4% of 1027	AFB1	1.1 to 32.9	HPLC-DAD	[118]
Rice	Brazil	11.2% of 187	AFB1	63.32	HPLC-FLD	[119]
Sorghum	Ethiopia	94.4% of 90	AFB1	33.1	ELISA	[120]
Spices, herbs, and nuts	Lebanon	100, 20.4, and 98.6% of 198	AFB1	0.97, 0.27, and 0.40	HPLC	[121]
Vegetable oil	Sri Lanka	34%	Sum of AFs	4.0	HPLC-FLD	[122]

### 4.2. OTA Occurrence

OTs represent another class of mycotoxins posing a significant threat to human and animal health due to their widespread presence in various food commodities [123]. Cereals like barley, rye, wheat, maize, sorghum, and oats serve as primary reservoirs for these toxins. Additionally, OTs contaminate coffee beans and grapes, persisting even in their processed forms such as wine and raisins [123]. For instance, de Andrade Silva et al. [124] introduce a groundbreaking detection method for OTA in coffee samples utilizing a spoon-shaped waveguide immunosensor. This innovative biosensor, featuring a surface covered with a 60 nm layer of gold to facilitate the surface plasmon resonance (SPR) phenomenon, demonstrated a linear correlation between SPR values and OTA concentration within the range of 0.2 ppt to 5 ppt. Notably, the biosensor exhibited exceptional selectivity and resistance to matrix interference when tested in coffee samples. The device, characterized by its portability, simplicity, and suitability for onsite detection, represents a significant advancement in coffee quality analysis. With a linear range spanning from 2 × 10^−4^ ppb to 5 × 10^−3^ ppb, the immunosensor demonstrated remarkable selectivity in OTA detection, distinguishing it from structurally similar compounds like OTB, glycose, and caffeine. Evaluation in soluble coffee samples further underscored the biosensor’s resilience to matrix interference, reaffirming its potential as an indispensable tool for real-time quality assessment in coffee production, devoid of the need for microfluidic systems. Moreover, Kochman et al. [124] investigated the concentrations of OTA, DON, T-2, and HT-2 toxins in dry red wines, sampling 19 wines from Spain, France, and Poland, representing both conventional and organic viticulture. Using ELISA, they found all wines exceeded regulatory limits for mycotoxin levels, with OTA and DON contamination varying by country of origin and farming method. Interestingly, T-2 and HT-2 toxin levels showed a negative correlation with pH. These findings suggest wine may be a significant source of mycotoxin exposure in the human diet, warranting further research to establish reference values and implement effective control measures to mitigate potential health risks.

Contamination can also occur in cottonseed, nuts, dried beans, and specific meat products, particularly those derived from porcine kidneys. Processed meats like sausages, bacon, and ham may also harbor OTs [125].

Barley and dry-cured sausages warrant specific attention as scientific studies have documented a high prevalence of OTA contamination in these food items [126,127]. Contamination in barley can transpire during various stages: growth in the field, storage, or subsequent processing (Table 5 is a compilation of recent studies on OTA contamination in diverse commodities). Dry-cured sausages, on the other hand, can become contaminated during the production process. Altafini et al. [128] conducted a comprehensive analysis of 172 different salamis sourced from farms and small-scale salami factories across four Italian regions, namely Piedmont, Veneto, Calabria, and Sicily, to assess the presence of OTA. Utilizing HPLC-FLD, the study established a detection limit (LOD) of 0.05 μg/kg and a quantitation limit (LOQ) of 0.20 μg/kg, with an average recovery rate of 89.1%. OTA was identified in 22 salamis, with 3 samples surpassing the Italian guidance value for OTA in pork meat (1 μg/kg). Notably, a significant proportion of positive samples were found among spicy salamis (68.2%), despite their relatively low representation in the overall sample pool. This observation suggests a potential link between red chili pepper contamination by OTA and the presence of the mycotoxin in these products. Consequently, rigorous control measures for ingredients used in salami production, particularly spices, are imperative. Therefore, while the occurrence of OTA in salamis remains relatively low, the prevalence of positive samples among spicy varieties underscores the importance of scrutinizing ingredient quality to mitigate mycotoxin contamination risks in meat products.

The extensive use of barley and dry-cured sausages as ingredients necessitates stringent monitoring practices throughout the food chain to ensure acceptable OTA levels and safeguard public health. Ochratoxin contamination exhibits a pronounced geographical distribution, with a higher prevalence observed in temperate regions. This can be attributed to the optimal environmental conditions for fungal growth, which thrive in cool temperatures around 24 °C and moderate moisture content ranging from 19–22% [129]. Consequently, countries in Northern Europe, the Balkans, and Canada face a greater risk due to these prevailing climatic conditions.

**Table 5:** Reports of ochratoxin A occurrence in food commodities over the last ten years.

Food	Country	Percentage of Contaminated Samples	Concentration (μg/kg)	Method of Detection	Reference
Baby foods	Turkey	34.7% of 150	<0.5	HPLC	[130]
Barley	Egypt	20% of 15	1.13–2.15	HPLC-FLD	[131]
Barley	United States	6% of 60	0.16–185.24	HPLC-FLD	[132]
Beers	Czech Republic	81% of 132	0.001–0.195	UPLC-FLD	[133]
Beer	Portugal	10.6% of 84	<0.43–11.25	HPLC-FLD	[134]
Beer	Spain	20% of 40	0.24 to 54.76	HPLC-MS/MS	[135]
Breakfast cereals	Serbia	33.7% of 136	0.07–3.00	HPLC-FLD	[136]
Cereals	Uganda	8.3 to 100% of 105	0.1–16.4	ELISA	[137]
Cocoa bean	Brazil	22.8% of 123	0.25–7.2	HPLC-FLD	[138]
Coffee	Portugal	25% of 6	1.45–1031	HPLC-FLD	[139]
Cheese	Italy	26.3% of 57	1.7–7.2	HPLC-MS/MS	[140]
Dried fruits	Morocco	17.1% of 210	0.8–99.1	HPLC-FLD	[141]
Dried grapes	Iran	57.5% of 23	0.16–8.40	HPLC-FLD	[142]
Dry-Cured Meat	Croatia	19.2% of 250	0.24–4.81	HPLC-MS/MS	[143]
Dry wine	Serbia	52.2% of 113	0.026	HPLC-FLD	[144]
Fermented coffees	Brazil	21.4% of 14	<0.64–0.87	HPLC-FLD	[145]
Pasteurized Milk	China	25.8% of 120	>0.049–18.8	HPLC-MS/MS	[146]
Maize	China	1.6% of 426	0–5	UPLC-MS/MS	[147]
Maize	Pakistan	71.0% of 46	2.14–214	HPLC	[148]
Milk	Italy	36.4% of 33	<0.3–3	HPLC/FLD	[149]
Rice	Portugal	2% of 36	1.9–2.2	ELISA	[150]
Rice Bran and Maize	Southeast Asia	99% of 125	43.7	LC-MS/MS	[151]
Raisin	USA	93% of 40	0·06–11·4	HPLC	[152]
Salamis	Italy	12.8% of 172	0.07–5.66	HPLC-FLD	[128]
Sorghum	Tunisia	24 of 064	1.04–27.8	HPLC-FLD	[153]
Wheat	United States	13% of 58	0.17–14.94	HPLC-FLD	[132]
Wine	Croatia	92.8% of 110	0.003–0.163	HPLC-FLD	[154]
Wine	Italy	71.9% of 57	Mean of 0.13	HPLC-FLD	[155]

Table 5 presents a diversity of data regarding the presence of OTA in a variety of foods and beverages across different countries. First, it is observed that samples of alcoholic beverages, such as beers and wines, showed a significant percentage of contaminated samples, with values as high as 81% in the case of the Czech Republic and 92.8% in Croatia. This finding suggests a potential food safety concern related to the presence of mycotoxins in fermented products. On the other hand, cases such as pasteurized milk in China, where 25.8% of samples showed contamination, raise questions about the effectiveness of pasteurization processes in eliminating mycotoxins. Additionally, the presence of OTA in staple foods such as cereals (including barley, maize, and wheat) and dairy products (milk and salami) highlights the importance of continuous surveillance in the food chain. The detection method used varies between HPLC, HPLC-FLD, HPLC-MS/MS, and ELISA, reflecting the need for sensitive and specific analytical methods for the precise detection of mycotoxins in different food matrices. Furthermore, the concentration ranges of OTA found in the samples were quite wide, underscoring the variability in contamination and the importance of regularly monitoring OTA levels in food to ensure food safety and public health.

The occurrence of AFB1 and OTA in food shares both similarities and differences. Both toxins are commonly found in various food products worldwide, including cereals, legumes, nuts, spices, beers, wines, milk, and meats, due to inadequate drying and storage practices [156,157,158]. They are also known to co-contaminate agricultural products, with a significant proportion of samples reported to be concurrently contaminated with both mycotoxins [157,158]. However, the specific environmental conditions and fungal species responsible for their production may vary, leading to differences in their prevalence in different food products and regions. Additionally, while both toxins are highly toxic and carcinogenic, OTA is considered the most toxic of the OTs, with a chlorinated structure that makes it more potent than AFB1 [157].

## 5. Effect of AFs on Human and Animal Health

AFs exert various detrimental effects on human and animal health, which can be acute or chronic depending on the dose and duration of exposure [159]. Humans typically ingest AFs through contaminated food, while animals are exposed through contaminated feed [160]. Table 6 summarizes the common fungal species producing AFs and OTA, the frequently contaminated food items, and their associated health effects.

The toxic potential of AFs, particularly AFB1, hinges on both dose and exposure duration. Other factors such as age, sex, species-specific tolerance, and nutritional status also play a crucial role. While the detrimental effects on animals are well-documented, human cases typically arise from accidental acute exposures or chronic consumption in regions with high levels of mycotoxin contamination [159].

Particularly in young chickens, studies have demonstrated that AFB1 exhibited the most potent toxicity, causing liver damage (hepatotoxicity), liver cancer (hepatocarcinogenicity), mutations (mutagenicity), and birth defects (teratogenicity) in various animals [161]. Furthermore, the flesh and other products derived from animals fed AF-contaminated feed may harbor AFs and their metabolites, posing a potential health risk to consumers [162].

The liver represents the primary target organ for AFs. Hepatic metabolism converts AFs into highly unstable molecules, which interact with liver cell macromolecules. Excessive interaction leads to cell death, manifesting as acute aflatoxicosis symptoms: jaundice, hemorrhagic liver necrosis, and hepatic encephalopathy (often fatal) [163]. Adults exhibit greater tolerance due to faster cell renewal rates [164].

AFs also suppress the immune system, increasing susceptibility to infections, which is particularly concerning in regions with prevalent diseases like HIV and malaria. In children, aflatoxin exposure can result in stunted growth and developmental issues due to its impact on nutrition and immune function [165].

**Table 6:** Main associated fungi producers of AFs and OTA, the frequently contaminated foods, and primordial toxic effects.

Mycotoxins	Main Producers	Food	Toxicity	Reference
Aflatoxins B1, B2, G1, and G2	*Aspergillus flavus* *A. parasiticus* *A. nomius*	Maize, peanuts, wheat, cottonseed, nuts, rice, dry fruits, and spices.	Carcinogenicity Genotoxicity Hepatotoxicity Immunotoxicity Teratogenicity	[166]
Ochratoxin A	*Penicillium verrucosum* *A. ochraceus* *A. carbonarius*	Cereals, cocoa, coffee beans, wine, grape juice, beer, spices, cured meat products.	Hepatotoxicity Immunotoxicity Nephrotoxicity Teratogenicity	[167]

Aflatoxicosis in poultry manifests as mortality, reduced growth, poor feed conversion, decreased egg production, lethargy, inappetence, fatty liver, and pigmentation issues [168]. Birds consuming contaminated feed become immunosuppressed and more susceptible to stress and diseases.

Aflatoxicosis causes significant pathological changes: increased liver size, paleness, enlarged bile ducts, and potential lesions in kidney and spleen tissues. Histopathological examination reveals vacuolar degeneration, fatty changes, hepatocyte degeneration, along with lymphocyte infiltration and bile duct hyperplasia [168]. Furthermore, aflatoxin exhibits carcinogenic properties in poultry.

AFB1, the most potent aflatoxin, is metabolized in the liver to aflatoxin B1-8,9-epoxide, which binds to DNA and forms mutagenic adducts. These mutations can lead to liver cancer, with a higher risk in birds exposed during early life stages [169]. Gholami-Ahangaran et al. [170] investigated the use of a commercial nano-compound in feed to mitigate aflatoxicosis in broiler chickens. While no performance improvement was observed with Nanocid in a regular diet, its addition to an aflatoxin-containing diet (3 ppm) significantly increased weight gain and feed intake, and improved feed conversion during the final two weeks.

**Figure 4:**
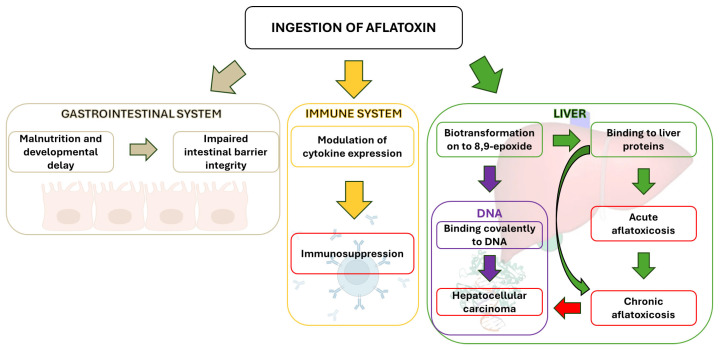
Different physiological levels of AFB1 toxicity.

While primarily affecting the liver, AFB1 can also impact other physiological processes (Figure 4). However, its hepatotoxicity and the interaction of its epoxide derivative with liver cell DNA remain the primary concerns. This interaction is linked to liver tumor development following chronic exposure to low AFB1 levels.

Classified as a Group 1 human carcinogen by the IARC in 1993, AFB1 is the most potent naturally occurring carcinogen. This classification is based on established links between AFB1 exposure and the development of liver cancer (hepatocellular carcinoma—HPC)—the most common global cancer and the third leading cause of cancer-related mortality [171].

AFB1 undergoes liver metabolism by CYP1A2 and CYP3A4 enzymes, leading to the formation of AFB1-8,9-endo-epoxide and the more prevalent and reactive AFB1-8,9-exo-epoxide. The latter can intercalate between DNA bases, forming mutagenic AFB1-N7-Gua adducts. In humans, p53 gene mutations (codon 249), activation of mitotic recombination, and minisatellite rearrangements contribute to liver tumorigenesis and HPC development [172].

Beyond its carcinogenic properties, AFB1 exhibits immunosuppressive effects, hindering the body’s defense against microorganisms. It impairs B and T lymphocyte activity and suppresses inflammatory cytokine production, potentially leading to persistent infections and posing a significant risk to immunocompromised individuals [69]. For instance, aflatoxin-albumin adducts exacerbate immune system damage in HIV-positive patients [165].

Aflatoxin exposure can also affect the gastrointestinal tract. Studies suggest a link between AFB1 and developmental delays in infants [173]. The frequent occurrence of malnutrition in developing countries may exacerbate aflatoxin’s effects, as protein deficiency disrupts liver detoxification enzymes, promoting toxin accumulation. Aflatoxin exposure is known to impair animal development and cause enterocyte damage, contributing to a leaky gut [174].

## 6. Effects of Ochratoxin on Human and Animal Health

The similarity in toxic effects of OTA and ochratoxin C is significant, even though OTA is more commonly found. In contrast, the potency of ochratoxin B is considerably lower [175]. Human and animal exposure to OTs is notably high, with detections in serum, plasma, and milk. Although concentrations of OTs in grains may vary, at times they can reach levels capable of causing illness [176].

The process of OTA toxicity involves its attachment to plasma proteins, specifically albumin, which helps transport it to the kidneys, liver, and other tissues [177]. OTA has also been found to hinder protein synthesis and mitochondrial respiration, cause DNA damage, and modify gene expression in various cell types such as renal cells, hepatocytes, and lymphocytes [178].

OTA is well-known for its toxic effects, particularly on the kidneys. It primarily targets the epithelial cells of the proximal tubules and can cause varying degrees of nephrotoxicity depending on factors such as dose, duration of exposure, age, sex, and genetic susceptibility. Research in animals has demonstrated that long-term OTA exposure leads to its accumulation in the renal cortex, resulting in oxidative stress, inflammation, apoptosis, and necrosis of renal cells with consequent dysfunction and fibrosis. Short-term exposure to OTA can also induce acute renal failure [179].

Exposure to OTA has been associated with a specific form of chronic kidney disease known as Balkan Endemic Nephropathy in humans. BEN is a progressive disease that affects people living in certain regions of the Balkans, and it is believed to be caused by long-term exposure to contaminated food and water sources [180]. In animals, OTA is highly toxic to broilers, pigs, dogs, and rats; it primarily functions as a nephrotoxin leading to severe nephrotoxicity. Among them all, pigs are the most sensitive to its effects. Other toxicities include myelotoxicities in mice, hepatic lesions, and cardiac issues in rats [160]. Animals exposed to OTA may experience reduced growth, poor body weight gain, as well as behavioral depression [181].

OTA also causes severe immunosuppression, resulting in teratogenic, mutagenic, and immunotoxic effects that increase mortality [181]. In animal studies, OTA has been shown to reduce the production of antibodies and the activity of immune cells such as lymphocytes and macrophages [182]. In humans, this mycotoxin has been described as a modulator of humoral and cellular immunity, inflammation, nitrosative stress, and gut immunity, which can increase the risk of infections and cancer [183].

Regarding OTA carcinogenicity, animal studies have shown that long-term exposure can increase the incidence of tumors, including kidney and liver tumors [184]. In humans, this mycotoxin has been associated with an increased risk of urinary tract tumors, as well as other types of cancer such as breast cancer [185]. In pregnant mice, OTA contamination levels of 20 ppm resulted in significant embryotoxic and teratogenic effects between day 7 and day 12 of pregnancy. A protective effect against these effects was observed with phenylalanine at 20 ppm in the diet [186]. Additionally, OTA has been found to have various adverse health effects including inhibition of protein synthesis, induction of DNA damage, potential long-term neuronal effects leading to Alzheimer’s disease, as well as nephrotoxic, hepatotoxic, immunotoxic, and carcinogenic impacts in animals and humans [187].

Investigations into the toxic effects of OTA on retinal ganglion cells have shown that it can cause oxidative stress, mitochondrial dysfunction, and apoptosis, leading to retinal damage. This includes an increase in reactive oxygen species and disruptions in the balance of antioxidant defenses, ultimately affecting the survival of retinal ganglion cells and visual function [187].

Microscopic alterations within organs due to OTA exposure include degeneration of liver tissue and proliferation of epithelium of the biliary channel, resulting in individual cell necrosis and DNA adduct formation. In kidneys, OTA causes necrosis of tubular epithelial cells, glomerular infiltration, and distended glomerular spaces. OTA also causes atrophy of follicles and a reduction in the lymphocytic mass present in the medulla within the bursa. Within the thymus, OTA exposure results in the reduction of lymphocytic mass in the parenchyma and localized congested areas [169].

In addition to the well-established nephrotoxicity, chronic exposure to OTA has been linked to the suppression of the immune system, increased risk of cancer, and other toxic effects, such as reproductive and neurological toxicity. While the mechanism of OTA toxicity is not fully understood, it is thought to involve the inhibition of protein synthesis in cells. Further research is necessary to better understand the detailed mechanisms of toxicity of OTA in both human and animal health.

## 7. Recent Outbreaks

Populations most at risk of mycotoxin contamination come from regions with inadequate regulatory enforcement and primary prevention measures. Recent outbreaks have predominantly occurred in tropical and subtropical areas, though climate change has also increased vulnerability in Mediterranean regions, influencing mycotoxin patterns due to changes in temperature, CO_2_ levels, and rainfall [188]. This global rise in mycotoxin contamination has necessitated the development of more precise analytical methods, such as advanced chromatographic and sensor-based techniques, to detect these toxins and aid in outbreak prevention [189].

Significant mycotoxin outbreaks include:

Kenya (2004): A severe outbreak of aflatoxicosis in rural Kenya resulted in 317 cases and 125 deaths, attributed to contaminated maize during a period of food scarcity exacerbated by drought [190].

Brazil (2011): An outbreak affected 65 dogs on 9 farms, leading to 60 deaths due to aflatoxin-contaminated maize in their diets. This event highlighted the risk to both livestock and pets from mycotoxin exposure [191]

Tanzania (2016): An unfamiliar disease affected various demographics, resulting in 68 cases and 20 deaths. Locally produced maize was identified as the source, emphasizing the need for vigilant monitoring of food products [192].

These incidents underscore the ongoing challenge of mycotoxin management in food safety. They reveal the critical need for comprehensive surveillance systems and effective regulatory frameworks to mitigate risks, particularly in vulnerable regions. This section highlights the importance of integrating modern detection methods with traditional food safety practices to enhance global food security and prevent future outbreaks.

## 8. Legislation

The frequent intake of AFs and OTA has led to serious health issues in both people and animals. To protect consumers from these harmful toxins, regulations establish maximum acceptable levels for OTA in various food categories. This global concern has prompted many countries to impose strict regulations on these mycotoxins, aiming to enhance the quality of commercial products and safeguard public health [193]. However, these standards can have a double-edged sword effect. While they enhance safety, they can also restrict trade from regions prone to contamination and potentially decrease the economic value of certain products if contamination is found [193]. It is important to recognize that socio-economic factors, such as inadequate government policies, can also contribute to conditions that favor mycotoxin contamination [194].

More than 100 countries have regulations on aflatoxin, with the primary goal of protecting human and animal health. However, these regulations also bring economic burdens to nations trying to export aflatoxin-contaminated products. It is important to consider both the financial impact and the regulatory benefits of these laws. It is crucial to note that even in countries with laws against mycotoxins, many people consume uninspected crops, especially in areas with extensive subsistence farming. This situation can lead to contamination, exposure, and lack of control, and consequently have adverse effects on global trade and public health [195].

The allowable limits for AFs in food intended for human consumption worldwide generally range from 2 to 30 μg/kg. The European Union has established stricter regulations for AFB1 and total AFs in directly consumed products, permitting up to 2 μg/kg and 4 μg/kg, respectively. In contrast, the US sets a maximum level of 10 μg/kg for both AFB1 and total AFs in all finished products. However, OTA is considered less toxic, with permissible levels ranging from 2 to 10 μg/kg in these regions.

In Brazil, the ANVISA’s RDC nº 722, as of 1 July 2022, establishes the maximum acceptable levels of mycotoxins in food. Similarly, regulations from the FDA in the US and those from the European Commission (EC No. 165/2010 and 2022/1370) determine these limits for maize and its derived products. Additionally, the maximum and recommended levels of AFs and OTA in feeds in the European Union are stipulated by Directive 2002/32/EC and Recommendation 2006/576/EC, respectively. While tolerable levels are generally higher in Brazil, each new regulation leads to increasingly stringent legislation. Table 7 presents a summary comparing the acceptable limits of AFs and OTA in foods between Brazil, the EU, and the US. It is noteworthy that currently there are no specific regulatory guidelines for OTA set by the FDA regarding its presence in food or feed within the US.

## 9. Methods for Avoiding or Mitigating the Presence of Mycotoxins in Foods

The most effective approach to reducing mycotoxins in the food chain is by preventing the growth of fungi in food and inhibiting toxin production. Different physical, chemical, and biological methods have been used at both industrial and laboratory levels to achieve this goal [196].

Numerous steps can be implemented to reduce the risk of mycotoxin exposure and related health and socio-economic challenges. These measures may involve preventing contamination by limiting fungal growth or intervening after growth to eliminate or reduce toxin availability. Pre-harvest treatments are primarily focused on controlling fungal infection spread in the field, while post-harvest methods aim at decontaminating substrates after toxin production or reducing toxin absorption by exposed organisms [123].

Cereal grains and feed are inevitably prone to contamination, with a lack of cost-effective detoxification methods. Hence, it is crucial to regularly monitor the quality of animal-derived foods, raw materials, and feed for the well-being of food animals, economic sustainability, and consumer food safety [5].

### 9.1. Good Agricultural Practice

The first critical point in limiting the occurrence of *Aspergillus* or *Penicillium* isolates and their mycotoxin contamination is the adoption of agricultural practices that can create an unfavorable environment for the proliferation of fungal spores present in the soil. These practices include plowing the soil before sowing, weeding, respecting the specific sowing time for each type of crop and the optimal harvest time, manuring, soil amendment and fertilization, irrigation management, and crop rotation with crops less susceptible to the growth of *Aspergillus* spp. [197].

Mycotoxin contamination can be minimized by certain cultural practices, curing, drying, and storage methods. However, these techniques may be incompatible with small-scale agriculture in emerging countries, particularly in tropical regions [198]. Thus, the development of mycotoxin-resistant varieties is a multi-step process that may involve direct selection for resistance to FG and aflatoxin formation, indirect selection for resistance or tolerance to biotic factors or environmental stresses, or selection for morphological characteristics that inhibit or delay fungal invasion or growth [197]. Due to the scarcity of resistance genes, the development of cultivars resistant to preharvest mycotoxin contamination has been limited. Numerous efforts have been made to produce mycotoxin-resistant cultivars, resulting in the creation of selected resistant types that have gradually been released as improved germplasm in several regions of the world. However, complete resistance to mycotoxin contamination has not been achieved and genetic efforts continue [199].

Similarly, mechanical damage should be avoided as it increases the grain’s susceptibility to fungal and insect attacks. For example, it is preferable to harvest maize cobs with their leaves intact and avoid damaging the leaves, as they are critical in protecting the cobs from insects, particularly weevils such as *Sitophilus zeamais*, which are the most common pests of maize crops. Not only can these insects increase the surface area of the ear susceptible to fungal infection, but their metabolic activity can also wet the grain and promote fungal growth [200]. Mechanical damage caused by milling should also be minimized as it facilitates insect penetration. Even if climatic conditions are not optimal, grain lesions caused by insect infestation can lead to mycotoxin contamination of the grain [201]. The fungi may then grow inside the grain, where they are isolated from environmental conditions and in direct contact with nutrients, creating a micro-atmosphere [202].

Harvesting at the optimal time is also critical to avoid fungal growth. Harvesting should occur shortly after physiological maturity to minimize mycotoxin contamination. Crops harvested at immature stages, on the other hand, must be dried promptly and effectively to achieve moisture levels that are no longer conducive to mold growth (10–13% for cereals), thus preventing mold development throughout the storage period [203].

Climatic conditions in many developing countries, which often combine inadequate early drying with excessive humidity, play a significant role in crop contamination and the often-high mycotoxin levels observed in agricultural commodities. In addition, although prolonged drying in the field may reduce grain moisture, this technique increases the time exposed to insect attack, resulting in increased losses during storage [204]. Therefore, unfortunately, the implementation of these storage and drying practices is often difficult for farmers with small plots of land, especially when the general climatic conditions are unfavorable (tropical and subtropical regions). To make matters worse, the effect of crop rotation and most agricultural practices on toxicity is generally more limited than the effect of environmental factors (temperature and humidity).

### 9.2. Chemical Approaches

Synthetic antifungal agents represent chemical compounds engineered to inhibit fungal growth and proliferation. They find widespread application in the preservation of food items, aimed at averting spoilage and enhancing their shelf life [205]. Despite their evident utility, synthetic antifungal agents are accompanied by certain drawbacks warranting critical consideration [205].

**Figure 5:**
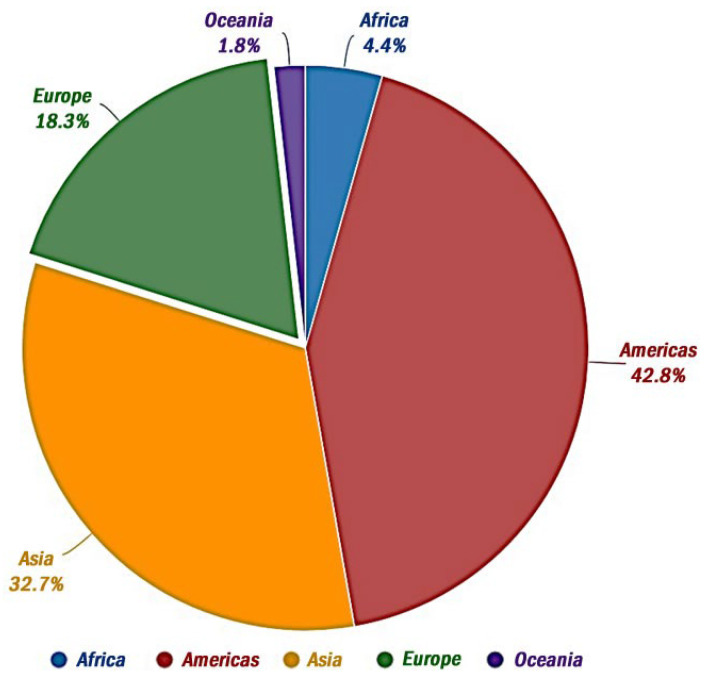
Total use of fungicides in continents between 1990–2021 (FAOSTAT, 2024).

One primary advantage associated with fungicides lies in their efficacy. Engineered to exhibit potent activity, they afford prolonged protection against a diverse spectrum of fungal species. Notably, synthetic fungicides offer effective mitigation against food contamination while also being relatively economical and user-friendly, rendering them a preferred choice among food manufacturers. Consequently, their adoption has become pervasive [206]. The utilization of fungicides for such purposes was most pronounced in Europe during the period spanning 1990–2021, as depicted in Figure 5.

Moreover, fungicides offer greater convenience compared to their natural counterparts. They can be produced in large quantities and transported and stored with ease, without the threat of spoilage. This is attributed to their suitability for food producers, requiring swift and efficient treatment of sizable food volumes, unlike biopesticides, which hinge on the availability of plant sources [207].

Despite their myriad benefits, synthetic antifungal substances are not devoid of drawbacks. This approach appears to be nearing its thresholds due to several factors: environmental contamination and adverse effects on animal and plant biodiversity, diminishing efficacy owing to the development of resistance among microbial populations, and the inevitable toxicity of these substances upon chronic exposure in animals. A primary concern revolves around their potential repercussions on human health and the environment. Certain synthetic antifungal substances have exhibited toxic effects, raising concerns about residue persistence on treated food items [208].

The global population, projected to reach 9.2 billion by 2050 with an annual growth of 70 million people, necessitates a 70% surge in food production demand. This surge primarily stems from evolving dietary patterns in emerging economies, characterized by heightened consumption of higher-quality foods such as meat and dairy products, and increased utilization of grains for animal feed. Expanding agricultural land is a challenge, with potential ramifications including damage to forests and natural ecosystems, which serve as natural adversaries to wildlife, crops, and crop pests. Additionally, agricultural land may be diverted towards the production of bio-based raw materials like biofuels or fibers rather than food [209,210].

Hence, there is a pressing need to enhance food production efficiency by reducing land, water, energy, fertilizer, and pesticide usage. This imperative assumes greater significance given the constraints faced. To ensure sustainable production, it is imperative to address the challenge of minimizing yield losses attributable to pests in agricultural settings [210].

When fungicides are applied to plants or products, they can disrupt the cell membrane of fungal pathogens or impede crucial cellular processes. In some instances, fungicides serve as preventive measures by establishing a protective barrier that immobilizes toxigenic fungi, curbing or preventing their colonization of the plant [211]. However, exercising caution in fungicide usage is paramount due to its potential impact on human health and the environment, coupled with the risk of fostering resistant fungal strains [212]. Striking a balance between the benefits and risks associated with synthetic antifungal substances in food production is paramount.

Regarding mycotoxin detoxification, various chemical agents, including acids, bases, reducing agents, and oxidizing agents, have been employed to transform mycotoxins into less toxic derivatives through structural alteration. Among these, ozone and ammonia stand out as extensively studied chemical detoxification treatments [213].

Ozone finds application in disinfecting vegetables, fruits, cereals, and mycotoxin detoxification processes [214]. The antifungal mechanism of ozone gas involves damaging the fungal membrane, enhancing mitochondrial degradation, inducing cytoplasmic disintegration, and promoting plasmolysis [215]. Furthermore, ozone has demonstrated efficacy in degrading AFs and OTA [216,217]. Oxidizing agents interact with functional groups within mycotoxin molecules, inducing changes in their molecular structures, resulting in the formation of products with reduced double bonds, molecular weight, and toxicity [218].

In addition to ozone, other bases like ammonia have been utilized to reduce several mycotoxins, including FUM, AFs, and OTs, to non-detectable levels [219]. However, the use of certain bases such as potassium hydroxide and sodium hydroxide may lead to undesirable and toxic reactions. Notably, seed treatment employing ammonia has been found effective in suppressing the growth of mycotoxigenic fungi [220].

### 9.3. Detoxifying by Physical Methods

Traditional decontamination methods for mycotoxins in food and feed involve various physical techniques utilized when preventive measures fail. These methods encompass several procedures such as dehulling, heating, plasma treatment, sorting and separation, radiation, immersion and washing, and adsorption. However, the efficacy of these techniques hinges on the extent of contamination and the distribution of mycotoxins within the product. Nevertheless, these methods may yield uncertain outcomes and can result in significant product losses [219].

Sorting, dehulling, or washing are typically employed as pre-processing methods. They serve as common approaches to eliminate low-quality particles from food and maintain food quality. For instance, cereal grains can be sorted based on various physical attributes such as density, color, shape, and size, while also identifying broken grains afflicted with fungal growth. Given the uneven distribution of mycotoxin contamination among grains, sorting, washing, or separating damaged food can markedly reduce the contamination levels [221]. Immersing and washing contaminated grains in water and discarding the floating fractions can generally eliminate some amounts of AFs and FUM. Furthermore, cleaning and scouring procedures, as highlighted by Milani and Heidari [222], can substantially diminish ochratoxin contamination in grains.

Research indicates that ionizing radiation, including gamma radiation, electron beams, or X-rays, presents a safe and effective alternative to chemical treatments for eradicating microorganisms from food and feed or reducing mycotoxin levels [223]. This technology, known as food irradiation, constitutes a physical-cold process widely adopted in the food industry across many nations. In particular, Khalil et al. [224] have demonstrated that gamma radiation effectively curtails the growth of *A. flavus* and *A. ochraceus*, thereby significantly reducing AFs and OTA levels by 33.3–61.1%, contingent upon the mycotoxin involved.

The efficacy of UV radiation varies depending on different conditions such as exposure time and wavelength. While UV radiation can stimulate sporulation and fungal growth in some cases, the incidence of shorter wavelengths has the opposite effect on biological organisms. García-Cela et al. [225] demonstrated the effectiveness of UV-B and UV-A against *A. carbonarius* and *A. parasiticus*, resulting in reduced production of OTA and AFs in a time-dependent manner.

Chemical methods employed for mycotoxin reduction may yield negative consequences, including alterations in nutritional value and palatability or the presence of toxic residues. Conversely, biological methods may be constrained by factors such as prolonged degradation time or incomplete degradation. Consequently, adsorption has emerged as a promising option for mycotoxin treatment [226].

Adsorption entails both chemical and physical forces, making it the most commonly utilized method to safeguard animals against mycotoxins. By employing a range of adsorbents such as clay, activated charcoal, and other modified polymers, mycotoxins can be effectively bound and immobilized, thereby reducing their toxic impact by preventing their absorption from the gastrointestinal tract [227]. However, selecting efficient adsorbents can be challenging since various mycotoxins may co-occur in foods, potentially amplifying their toxic effects through synergistic interactions [226]. Some chemicals exhibit weak interactions with mycotoxins due to their polarity, solubility, molecular size, shape, and surface area, facilitating adsorption between adsorbents and mycotoxins [228].

Despite its efficacy, concerns persist regarding the safety of adsorbent materials, the removal from feed, and the disposal of adsorption chemicals and adsorbent-mycotoxin complexes. Consequently, some chemical adsorbents have been prohibited as detoxification materials in the food industry by the European Union (EU) [229].

### 9.4. Biocontrol of Toxigenic Fungi and Biodegradation of Mycotoxins

In the literature, several conventional physical, chemical, and adsorption-related technologies have been reported for the elimination or inactivation of mycotoxins [230]. Unfortunately, these approaches suffer from drawbacks such as safety concerns, loss of nutritional value and palatability, limited effectiveness, and cost implications. Recent research indicates promising prospects for using mycotoxin-adsorbing compounds to bind mycotoxins in the gastrointestinal tract of animals, thereby reducing their bioavailability and toxicities, particularly in the feed industry. However, the effectiveness of various adsorption agents differs, with some being more beneficial in preventing aflatoxicosis than others [70,230]. Consequently, there is a pressing need for decontamination technologies that are efficient, practical, and environmentally friendly.

To address these challenges, biological control techniques have been developed to manage foodborne pathogens more effectively and rapidly. Biocontrol involves controlling pathogenic microorganisms or their derivatives using natural sources such as microorganisms, plant-derived fungicides, and detoxifying enzymes. This approach has gained traction due to its ease of application and cost-effectiveness, positioning biological management as an eco-friendly alternative to synthetic compounds [231].

Currently, bio-protective crops, ferments, and purified molecules with antifungal activity are being developed. Microbial strains with potential antifungal properties have been isolated from various sources, leading to increased food shelf life and decreased fungal contamination, particularly from *Aspergillus* and *Penicillium* as described by Salas et al. [205]. LAB are commonly employed for food biopreservation, while *Trichoderma* spp. plays a significant role in plant biocontrol, promoting growth and inducing defenses against biotic and abiotic stresses [232]. Additionally, microorganisms such as *Debaryomyces hansenii* yeast and *Penicillium* spp. fungi combat meat product decay caused by fungi [233,234].

Biodetoxification techniques offer another viable solution for managing mycotoxins, involving the use of microorganisms or enzymes to break down mycotoxins into non- or less harmful compounds [235]. This method may entail using live or dead microorganisms to bind toxins to their cell wall components or decompose them into less harmful substances [236]. However, if mycotoxins are only adsorbed and not completely degraded, there is a risk of their delayed release in the gastrointestinal tract [237]. Numerous studies have identified fungal and bacterial strains capable of effectively breaking down mycotoxins, although concerns about food quality and consumer acceptance of meals enhanced with microorganisms persist. Consequently, microorganisms employed in food and feed additives must meet specific criteria, including safety, nonpathogenicity, production of stable and non-toxic metabolites, proficiency in mycotoxin degradation, formation of irreversible complexes, activity during storage, absence of unpleasant odors or tastes, retention of nutritional value, and minimal cultivation and production efforts. While various microorganisms have been proposed as potential detoxifiers for food and feed, only a few have undergone thorough testing to determine their efficacy [238]. The use of mycotoxin-degrading enzymes produced by bacteria and fungi may overcome some of these constraints [239].

Recent advancements in computational biology and synthetic biology have significantly enhanced the identification and understanding of mycotoxin-degrading enzymes, crucial for biological control methods. According to Sandlin et al. [240], computational tools are increasingly utilized to unearth strains and enzymes capable of detoxifying mycotoxins, which are often underexplored due to the vast potential diversity and complexity of biological systems. They elaborate on leveraging computational biology for searching genomic databases to identify candidate organisms with potential mycotoxin detoxification capabilities, which can be further enhanced via synthetic biology techniques to optimize the expression and activity of these enzymes. For instance, Zhang et al. [241] utilized a positive unlabeled deep learning approach to identify new enzymes capable of degrading mycotoxins such as OTA, demonstrating the potential of machine learning to predict enzyme-substrate specificity in complex substrates traditionally challenging for experimental approaches. This method, leveraging data from extensive biochemical databases, underscores a shift towards rapid, high-throughput screening methods that could revolutionize the discovery of biodegrading enzymes in synthetic biology frameworks. Similarly, the work by Liu et al. [242], though primarily a correction notice, indirectly highlights the importance of precise genetic tools like CRISPR/Cas for engineering industrial fungi to degrade mycotoxins, reflecting the growing integration of synthetic biology in developing practical applications for mycotoxin mitigation. These studies collectively illustrate the cutting-edge integration of computational and synthetic biology techniques to accelerate the discovery and functional analysis of mycotoxin-degrading enzymes, paving the way for more effective biological control strategies.

#### 9.4.1. LAB as a Potential Biocontrol Agent

LAB comprise a group of oxygen-tolerant, Gram-positive bacteria pivotal in the fermentation of diverse food and beverage products. These bacteria are characterized by their ability to metabolize carbohydrates during fermentation, yielding lactic acid as a primary product, which contributes significantly to the sensory attributes such as flavor, texture, and aroma unique to each fermented product [243].

Traditionally, the core group of LAB consists of four genera: *Lactobacillus*, *Leuconostoc*, *Pediococcus*, and *Streptococcus*. Recent taxonomic revisions have expanded this group to include several new genera, including *Aerococcus*, *Alloiococcus*, *Carnobacterium*, *Dolosigranulum*, *Enterococcus*, *Globicatella*, *Lactococcus*, *Oenococcus*, *Tetragenococcus*, *Vagococcus*, and *Weissella* [244].

Classification within LAB is based on their fermentation characteristics, growth conditions, and ability to produce lactic acid. They can also be categorized as homofermentative or heterofermentative organisms based on their carbohydrate fermentation abilities. Homofermentative LAB, such as *Lactococcus* and *Streptococcus*, produce two lactate molecules from one glucose molecule, while heterofermentative LAB, such as *Leuconostoc*, *Weissella*, and some *lactobacilli*, generate lactate, ethanol, and carbon dioxide from glucose [245].

Recent taxonomic revisions have led to a significant overhaul of LAB classification due to the complexity within the original genus *Lactobacillus*. This complexity necessitated the reclassification of the genus into 25 genera, including the original *Lactobacillus*, *Paralactobacillus*, and 23 novel genera such as *Amylolactobacillus*, *Acetilactobacillus*, *Agrilactobacillus*, *Apilactobacillus*, *Bombilactobacillus*, *Companilactobacillus*, *Dellaglioa*, *Fructilactobacillus*, *Furfurilactobacillus*, *Holzapfelia*, *Lacticaseibacillus*, *Lactiplantibacillus*, *Lapidilactobacillus*, *Latilactobacillus*, *Lentilactobacillus*, *Levilactobacillus*, *Ligilactobacillus*, *Limosilactobacillus*, *Liquorilactobacillus*, *Loigolactobacilus*, *Paucilactobacillus*, *Schleiferilactobacillus*, and *Secundilactobacillus* [246].

Under the previous taxonomic classification, LAB species extensively studied for their antifungal properties belong to the genera *Lactobacillus*, *Pediococcus*, and *Leuconostoc* [247]. The use of LAB as a biopreservation strategy is favored over other organisms, especially considering their inclusion in the Qualified Presumption of Safety (QPS) list by the EU and their Generally Recognized As Safe (GRAS) status by the US Food and Drug Administration (FDA) [248].

**Figure 6:**
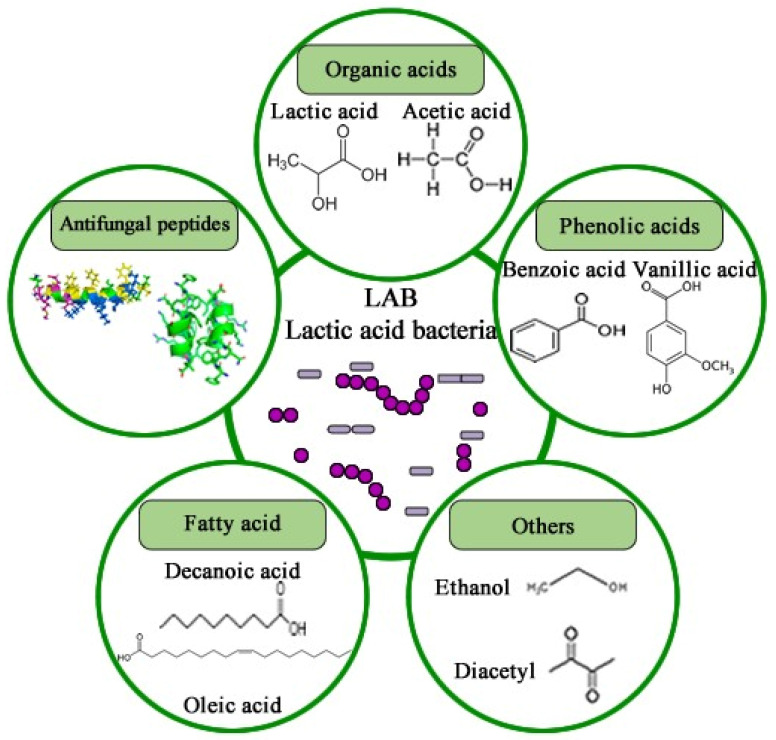
Chemical structure of antifungal compounds produced by LAB.

The inhibitory efficacy of LAB in food preservation is primarily attributed to the synthesis of metabolites during the fermentation process, with nutrient and space competition also recognized as contributing mechanisms [249]. Throughout fermentation, LAB produces a diverse array of antifungal metabolites, including organic acids, phenolic acids, volatile acids, CO_2_, hydrogen peroxide, antimicrobial peptides (AMPs), fatty acids, ethanol, and diacetyl, among others (Figure 6). These metabolites can exhibit synergistic or additive effects, complicating the precise elucidation of LAB’s antifungal mechanisms [250].

Organic acids such as lactic, acetic, and propionic acids exert antifungal effects primarily by disrupting the proton gradient essential for fungal cellular processes. Lactic acid, for instance, permeates the fungal cell membrane in a hydrophobic state, subsequently hydrolyzing within the cell to release H+ ions, inducing cytoplasmic acidification. Acetic and propionic acids similarly inhibit fungal amino acid absorption, albeit their efficacy is contingent upon the low pH environment created by lactic acid [251].

Antifungal peptides (AFPs), a subset of AMPs, are small, cationic peptides synthesized by LAB, capable of perturbing fungal membranes or interfering with proton gradients across the cell membrane. Notably, lactoferricin B demonstrates an affinity for the fungal surface, disrupting membrane integrity and exhibiting potent antifungal activity [252]. AFPs represent a focus of contemporary research into natural biological control agents, sourced from plant, animal, and microbial origins, and composed of amino acids linked via peptide bonds [253].

Numerous phenolic compounds have been identified in foods or media fermented with LAB, boasting varied properties encompassing antioxidant, antifungal, and antitoxigenic activities [254,255]. Key among the phenolic acids produced by LAB is phenyllactic acid and its derivative, 4-hydroxyphenylactic acid, recognized for their contribution to the antifungal activity of LAB-fermented media, thereby enhancing food shelf life [254,256]. Other phenolic acids generated during LAB fermentation include succinic acid, 4-hydroxybenzoic acid, vanillic acid, caffeic acid, p-coumaric acid, salicylic acid, ferulic acid, and benzoic acid [257].

#### 9.4.2. Detoxification of Mycotoxin by LAB

**Figure 7:**
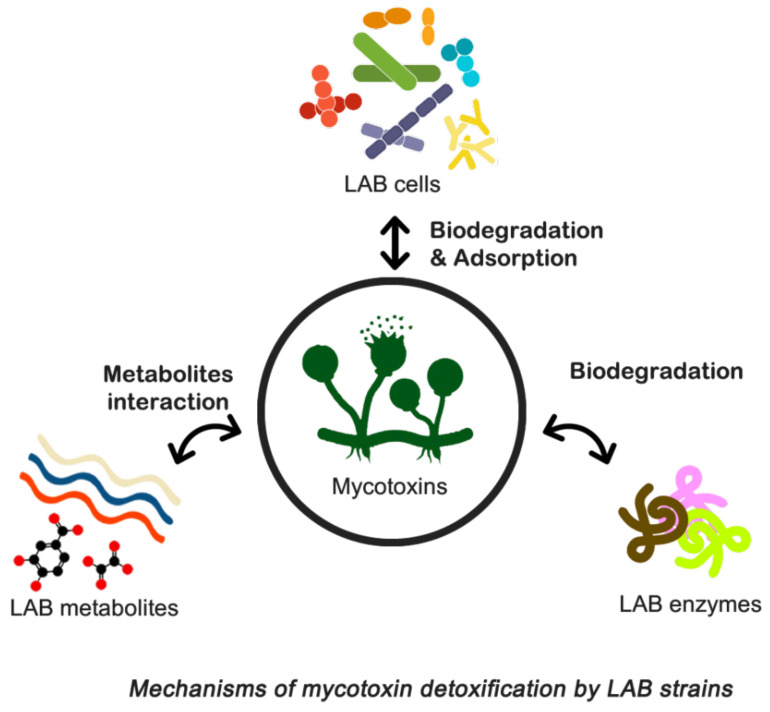
Three potential mechanisms of mycotoxin detoxification in foods by LAB.

The process of mycotoxin detoxification in foods facilitated by LAB involves three primary mechanisms: LAB enzyme degradation, adsorption by LAB cells, and interaction between mycotoxins and LAB metabolites (Figure 7). Proteolytic enzymes produced by LAB play a crucial role in this detoxification process [258]. Moreover, certain strains of LAB are believed to adsorb mycotoxins in specific foods, attributed to the composition of their cell wall containing polysaccharides, protein, and peptidoglycans [259]. However, the precise mechanisms underlying mycotoxin removal and degradation by LAB cells and metabolites remain elusive, with several hypotheses proposed, including degradation facilitated by proteolytic enzymes and specific metabolite binding to mycotoxins [259].

The study elaborated by Badji et al. [260] presented an insightful examination of the detoxifying capacity of LAB against two significant mycotoxins, AFB1 and OTA, often found in Algerian wheat products. Utilizing API 50 CHL and 16S rDNA sequencing methodologies, eleven LAB strains were proficiently identified and subsequently tested for their detoxification potential. The study’s findings revealed that both viable and heat-inactivated LAB cells, including LAB strains Lab-L4/al and Lab-L1 as well as the reference LAB strain *Lactobacillus plantarum* R1096, were effective in the reduction of AFB1 and OTA in vitro, albeit with different removal efficiencies among the strains. Notably, nonviable cells exhibited a superior capacity to detoxify AFB1. Furthermore, the detoxification efficiency of viable cells from Lab-L4/al and LP R1096 demonstrated a dependence on pH levels, with augmented removal at a lower pH; however, this pH sensitivity was not observable in the Lab-L1 strain. The study’s conclusions advocated the potential application of the examined LAB strains as fortifying agents in the fermentation of wheat-based foods, aiming to mitigate mycotoxin contamination and thereby enhance food safety.

Arun et al. [261] explored the capacity of LAB isolated from animal excreta to mitigate AFB1. Notably, the authors discerned that three out of fifty-six LAB isolates demonstrated over 50% sorption efficacy toward AFB1, highlighting the promising probiotic potential inherent in these strains: *Lactococcus lactis* strain CF_6, *Lacticaseibacillus casei* strain CW_3, and *Lactobacillus acidophilus* strain CE_4. The detoxification mechanism predominantly relied on the surface binding properties of the LAB, a critical feature that varies among strains, as elucidated through both biochemical analysis and Scanning Electron Microscope imagery. The study’s results suggested that while some isolates possessed notable AFB1 binding capacities, the efficacy was strain-specific, with the top three isolates displaying significant binding rates. Nonetheless, the authors’ findings were predicated on in vitro assays, underlining the necessity for further research to substantiate the applicability and scalability of such detoxification mechanisms within real-world food systems. These results indicated that, although the study provided an innovative perspective on leveraging probiotic LAB for AFB1 detoxification, thorough validation of in vivo conditions remains imperative for evaluating their practical efficacy and safety.

The study by Escrivá et al. [262] investigated the potential of *Lactobacillus* strains, specifically sourced from goat milk whey, on the biodegradation of two significant mycotoxins, AFB1 and OTA, during the bread-making process. Encouragingly, the research demonstrated that certain *Lactobacillus* strains can effectively reduce the levels of these mycotoxins, with *L. plantarum* B3 and *L*. *paracasei* B10 exhibiting the highest detoxification capabilities. During controlled fermentation, the incorporation of these lyophilized bacteria resulted in reductions of AFB1 and OTA by up to 27% and 32%, respectively, in the dough stage and up to 55% and 34% in the final bread product. While this indicates a promising biocontrol strategy for mitigating mycotoxin contamination in bread and bakery items, the study does not fully explore the mechanisms underlying such biotransformation. The findings suggested the potential for a cost-effective, large-scale detoxification protocol; however, further elucidation of the detoxification pathways and their efficacy in diverse bread-making environments would strengthen the application of this research to industry.

In another work, Dong et al. [263] undertook a rigorous examination of the detoxifying capabilities of two rumen-derived *Enterococcus* species on mycotoxin-contaminated corn silages. The authors adeptly characterized the mycotoxin degradation potential of *E. faecalis* and *E. faecium*, illustrating their pivotal role in the fermentative transformation and subsequent hygienic improvement of silage. The study revealed that *E. faeecium* exhibits a marked proficiency in reducing deoxynivalenol and AFB1 concentrations, outperforming *E. faecalis* in the latter’s eradication, yet displaying a lesser capability in zearalenone detoxification. Critically, the findings suggest that the inoculation of these *Enterococcus* species resulted in a notable alteration in microbial community dynamics, favoring a reduction in toxigenic fungal populations. Despite these promising results, the study failed to unravel the intricate molecular interactions between the microbial inoculants and the mycotoxigenic fungi, leaving a gap in our mechanistic understanding of the observed detoxification phenomena. Nonetheless, the authors have put forth compelling evidence supporting the deployment of these *Enterococcus* strains as a biologically based strategy to mitigate the health risks posed by mycotoxin contamination in feedstocks. These studies have contributed a valuable perspective to the field of bioremediation of food contaminants and underscore the need for continued exploration into the mechanisms and practical applications of mycotoxin detoxification by LAB.

### 9.5. Essential Oils

There is a current trend towards the utilization of natural compounds to enhance food safety, leading to extensive research on natural fungicides for the management of postharvest fungal disorders in agricultural produce. These natural phytosanitaries can be sourced from various origins, including microorganisms [264].

Zubrod et al. [211] highlighted that fungicides derived from plant-based products offer reduced environmental impact and pose lower risks to human health compared to conventional agrochemicals, largely due to their rapid degradation. Consequently, these fungicides contribute to both environmental preservation and consumer safety. One approach involving plant-derived compounds focuses on harnessing bioactive secondary metabolites, such as phenols, terpenes, aliphatic alcohols, aldehydes, ketones, and essential oils (EOs), which have been extensively studied. EOs, complex mixtures of volatile and lipophilic substances primarily obtained from plants via steam distillation [265], contain diverse molecules like terpenes and hydrocarbons, exhibiting significant in vitro antimicrobial potential. However, the practical application of EOs is somewhat limited due to the substantial doses required to achieve antimicrobial effects and potential flavor impacts on food products [266].

In recent years, oregano, cinnamon, thyme, rosemary, fennel, clove, and eucalyptus have emerged as the most utilized EOs in combatting mycotoxigenic fungi and their associated mycotoxins. The inhibitory effects of EOs on fungal growth and mycotoxin synthesis have been elucidated through various mechanisms, including modification of fungal growth rates and extension of lag phases, disruption of cell permeability, and modulation of gene expression patterns and metabolic processes linked to the electron transport chain [257].

Mustard, an herbaceous plant belonging to the *Cruciferae* or *Brassicaceae* family, encompasses various varieties, including white or yellow mustard (*Brassica hirta* or *Sinapis alba*), black or royal mustard (*Brassica nigra*), and oriental or brown mustard (*Brassica juncea*), all of which exhibit significant potential against fungal growth [267,268].

These plants are distinguished by the presence of secondary metabolites called glucosinolates (GTs), which emit a distinctive pungent odor upon hydrolysis [269]. GTs serve a pivotal role in plant defense mechanisms. Upon physical damage, plants are vulnerable to fungal infestations and other potential attacks, prompting the hydrolysis of these substances by the enzyme myrosinase, resulting in the production of isothiocyanates (ITCs) in the presence of water, among other byproducts [270].

ITCs possess multifaceted properties, including biocidal activity against fungi, bacteria, and insects [271], and their efficacy against fungal growth is well-documented [272]. Moreover, they exhibit herbicidal, antioxidant, and anticarcinogenic properties [273,274].

In the case of oriental mustard, the hydrolysis of glucosinolate sinigrin by myrosinase yields allyl isothiocyanate (AITC). Similarly, in yellow mustard, the hydrolysis of glucosinolate sinalbin by myrosinase produces p-hydroxybenzylisothiocyanate (p-HBIT). Both ITCs have been evaluated, demonstrating their capacity to inhibit fungal growth, as evidenced by studies on AITC [275] and p-HBIT [276].

Additionally, mustard contains other bioactive compounds, such as phenolic acids, renowned for their potent antioxidant activity. Plants utilize these compounds for growth promotion and resistance to pests and pathogens, among other functions [277].

AITC is a volatile compound associated with various beneficial effects on human health, including antiangiogenic, anti-inflammatory, neuroprotective, and anticarcinogenic properties [278]. AITC is the most studied and potent antimicrobial among ITCs, due to its antimicrobial action at lower doses Among ITCs, AITC is the most extensively studied and potent antimicrobial agent, exhibiting antimicrobial activity at lower doses [279]. Table 8 shows the antifungal activity of AITC against different fungal-toxigenic strains. Several studies have demonstrated AITC’s ability to volatilize and inhibit the growth of mycotoxigenic fungi, such as *F. graminearum*, *A. parasiticus*, *P. expansum*, and *F. poae*, with effects dependent on dosage and mitigating mycotoxin production starting at 10 μL/L in the gas phase [280,281,282]. Furthermore, AITC can directly react with mycotoxins, forming new compounds and reducing their presence in food solutions and matrices [283].

To investigate the potential of allyl isothiocyanate as an effective antifungal agent against *Candida albicans*, particularly its modes of action in combating drug resistance, Patil et al. [294] aimed to assess the efficacy of AITC in inhibiting ergosterol biosynthesis, inducing reactive oxygen species production, arresting the cell cycle, and affecting gene expression related to virulence factors in *C. albicans*. Furthermore, evaluating the anti-infective capability of AITC in an in vivo model using silkworms to demonstrate an increased survival rate against *C. albicans* infections. They examined a variety of potential therapeutic agents, giving particular attention to their detoxification mechanisms against the biofilms of *C. albicans*. Their investigation revealed that natural products, such as allyl isothiocyanate, piperine, and honokiol, exhibited promising results in impeding the biofilm formation and virulence of *C. albicans* by disrupting cell membrane integrity and inducing apoptosis. These findings are significant, as they highlight the potential of alternative, plant-derived compounds in addressing the challenges posed by fungal biofilms—an area that has been a substantial hurdle in clinical settings. The research delineated the specific modes of action of these agents, including the inhibition of ergosterol synthesis and the attenuation of pathogenicity through virulence factors. However, the author’s critical analysis underlines the necessity for further studies to better understand the clinical implications of these mechanisms, with a cautious tone suggesting the need for a translation from laboratory results to effective, safe, and clinically adopted antifungal therapies.

In the study carried out by Ren et al. [295], the authors explored the potential of horseradish oil and a selection of isothiocyanates as natural preservatives for postharvest tomatoes. The core investigation centered around the antifungal capacities of these agents against several pathogens known to cause decay in stored tomatoes. In particular, AITC demonstrated notable efficacy in suppressing mycelial growth on fungal agents such as *Botrytis cinerea*, *Alternaria alternata*, *Rhizopus stolonifer*, and *Geotrichum candidum*. The study’s results evidenced a commendable performance of certain ITCs in curbing tomato decay rates, weight loss, and maintaining fruit hardness, as well as slowing the reduction of acidity and total soluble solid content. However, the research does not explicitly investigate the detoxification mechanisms. It is evident that the study presents significant insights into alternative preservative methods and highlights the need for safer options to synthetic chemicals. Yet, the scope for elucidating the detoxification pathways and the complexity of interactions between these compounds and the biological system of the fruit remains an avenue for further in-depth research.

In another context, Hareyama et al. [296] assailed the pervasive challenge of aflatoxin contamination by investigating the antifungal efficacy of isothiocyanates. The study quantified the inhibitory impact of benzyl, allyl, methyl, and phenylethyl ITCs on both the growth of *A. flavus* and the production of the highly toxic metabolite AFB1. Among the observations, benzyl ITC emerges as a particularly potent growth inhibitor in its dissolved state, while AITC displayed its most pronounced impact in the gaseous state, underscoring the volatility and state of matter as crucial factors in the detoxification strategies of aflatoxigenic fungi. The persistence of benzyl ITC compared to the fleeting presence of AITC further accentuates the complexity of applying these compounds in practical scenarios for the effective mitigation of aflatoxin risks. This juxtaposition of efficacy across different states of matter adds a nuanced layer to the current understanding of ITCs’ potential as antifungal agents and begs a deeper exploration of application methods tailored to exploit the specific chemical stability and volatility profiles of individual isothiocyanates.

In the study elaborated by Li et al. [275], the authors explored the toxicity mechanisms of AITC on the soil-borne fungal pathogen *Fusarium solani*, implicating a molecular target for potential fungicide development. The authors demonstrated that AITC induced rapid fungal growth inhibition and morphological abnormalities while causing significant electrolyte leakage from fungal cells. They identified that the STRPC family member, FsYvc1, plays a critical role in *F. solani* response to AITC exposure. The genetic approach revealed that the absence of FsYvc1 in the fungi led to a heightened sensitivity to AITC and an increase in reactive oxygen species accumulation. The study further discussed the differential expression of glutathione-S-transferase, a detoxifying enzyme, noting that its levels raised substantially in both wild-type and FsYvc1-deficient strains upon AITC exposure, although no difference existed between these strains in the absence of AITC treatment. These findings provided essential insights into AITC’s antifungal action and suggested that targeting FsYvc1 could enhance the development of novel fungicides. However, the work would benefit from a broader examination of the potential ecological effects of AITC use and its implications on microbial communities within agricultural settings. In general, the previous works evaluated evidenced AITC as a good candidate as an alternative to chemical antifungal compounds.

Evaluating the control methodologies reviewed in the literature reveals that no single method effectively prevents and controls all mycotoxins; the most appropriate strategy depends on the specific mycotoxin, its characteristics, and the context of contamination. However, there are some general strategies and methods commonly used for controlling mycotoxins such as AFB1 and OTA. For AFs, physical methods including cleaning, separation of screenings, and washing have proven effective to a certain extent in reducing contamination levels. Additionally, biological methods that utilize LAB in food and feed can also help reduce exposure to dietary AFs. Concerning physical adsorption, adsorbents like activated charcoal, bentonite, and aluminosilicates have been researched for their capacity to bind AFs and reduce their bioavailability. However, the effectiveness of these adsorbents varies based on factors such as the type of adsorbent used, the concentration of aflatoxin, and the food matrix involved. For OTA biological control, methods using microorganisms like yeasts and LAB that can bind and adsorb OTA have been extensively studied. Chemical methods, including ozone treatment, have also shown potential in reducing OTA levels in various food commodities.

Overall, controlling mycotoxins typically involves a combination of physical, biological, and chemical methods, each tailored to the specific mycotoxin and the particular scenario of contamination. This multi-faceted approach ensures a comprehensive strategy for reducing the risks associated with mycotoxin contamination in food products.

## 10. Conclusions

AFs and OTs occur naturally in food and have detrimental effects on human and animal health, as well as causing economic losses. They are known to have carcinogenic, mutagenic, and estrogenic effects in humans. The main mycotoxins contaminating commodities are AFs and OTA to a degree of toxigenic significance, and most of them are produced during storage due to inappropriate conditions. To prevent the proliferation of mycotoxins, measures such as timely grain harvesting, proper drying, and good storage conditions are essential. In addition, the use of natural fungicides such as AITC and other biocontrol agents, together with grain processing methods, is important to reduce the concentration of these mycotoxins in foodstuffs. In this context, the inclusion of LAB can be beneficial, as their potential to inhibit the growth of mycotoxigenic fungi and reduce mycotoxin levels in food and feed has been studied. Therefore, the incorporation of LAB in storage and processing practices may be an effective strategy to mitigate the risk of mycotoxin contamination in food and feed.

Looking forward, significant advancements are anticipated in the realm of mycotoxin research and management, driven by technological innovation and interdisciplinary collaborations. The development of more precise and rapid detection methods remains a critical priority even though computational biology has come to improve the branch of mycotoxin detoxification. Innovations such as portable biosensors and machine learning-assisted diagnostic tools are expected to revolutionize the monitoring and management of mycotoxin contamination, enabling real-time detection at lower costs and with greater accuracy. Furthermore, there is a promising horizon for the development of genetically modified crops that are inherently resistant to mycotoxin-producing fungi, potentially reducing reliance on chemical fungicides and aligning with sustainable agricultural practices. The integration of climate-smart agricultural techniques is also anticipated to play a pivotal role in mitigating the impact of environmental changes on mycotoxin prevalence. These future developments not only hold the potential to enhance food safety and security globally but also aim to mitigate economic losses in the agriculture sector, thereby supporting the livelihoods of farmers and communities worldwide. Additionally, strengthening global collaborations and harmonizing regulatory standards can lead to more cohesive and effective international responses to the challenges posed by mycotoxins.

## Figures and Tables

**Table 1:** Degree of carcinogenicity of mycotoxins according to IARC.

Mycotoxin	Degree of Evidence of Carcinogenicity		Overall Evaluation of Carcinogenicity to Humans
	In Humans	In Animals	
Aflatoxin B1	Sufficient	Sufficient	Group 1
Aflatoxin B2		Limited	Group 1
Aflatoxin G1		Sufficient	Group 1
Aflatoxin G2		Inadequate	Group 1
Aflatoxin M1	Inadequate	Sufficient	Group 2B
Aflatoxin M2	-	-	Not classified
Ochratoxin A	Inadequate	Sufficient	Group 2B

Group 1, carcinogenic to humans; Group 2B, possibly carcinogenic to humans.

**Table 7:** Limit maximum tolerability of AFs and OTA in food commercialized in Brazil, the European Union (EU), and the United States (US).

Region	Food Category	AFB1 (µg/kg)	Sum of AFs (µg/kg)	AFM1 (µg/kg)	OTA (µg/kg)
Brazil	Cereal-based for infant	-	1.0	-	2.0
	Cocoa beans	-	10.0	-	10.0
	Peanuts, Brazil nuts	-	20.0	-	-
	Coffee	-	-	-	10.0
	Nuts, walnuts, pistachios, hazelnuts, and almonds	-	10.0	-	-
	Cereals and cereal products	-	5.0	-	10.0
	Maize and maize products	-	20.0	-	20.0
	Dehydrated and dried fruits	-	10.0	-	10.0
	Cocoa and chocolate	-	-	5.0	5.0
	Powdered milk	-	-	5.0	-
	milk	-	-	0.5	-
	Cheese	-	-	2.5	-
	Grape juice, grape, wine, and its derivatives	-	-	-	2.0
EU	Cereal-based for infant	0.10	-	-	0.5
	Peanuts and tree nuts	2.0	4.0	-	-
	Brazil nuts, hazelnuts	5.0	10.0	-	-
	Coffee	-	-	-	3.0 to 5.0
	Almonds, pistachios, and apricot kernel	8.0	10.0	-	-
	Cereals and cereal products	2.0	4.0		3.0 to 5.0
	Maize and maize products	5.0	10.0	-	-
	Dehydrated and dried fruits	2.0	4.0	-	2.0 to 8.0
	Cocoa powder	-	-	-	3.0
	Powdered milk	-	-	-	-
	Milk	-	-	0.05	-
	Infant milk	-	-	0.025	-
	Grape juice, grape, wine, and its derivatives	-	-	-	2.0
	Products for animal feeding	50	-	-	250
	Feed	5 to 50	-	-	10 to 100
US	Foods	-	20.0	-	-
	Brazil nuts	-	20.0	-	-
	Peanuts and Peanut products	-	20.0	-	-
	Pistachio nuts	-	20.0	-	-
	Milk	-	-	0.5	-
	Animal Feeds	-	20.0–300.0	-	-

**Table 8:** Recent reports about the use of AITC Antifungal to prevent fungal growth and extend the shelf life of foods.

Food or Primary Commodity	Application Mode	Dosage (µL/L)	Fungi Evaluated	Reference
Almonds	Hydroxyethyl-cellulose antifungal device and a paper filter containing AITC during storage (15d).	5.07, 10.13, and 20.26 mg/L	*A. flavus*	[284]
Barley	Hydroxyethyl-cellulose gel disk	50	*Penicillium* *verrucosum*	[285]
Barley	Paper filter containing AITC during storage (90d)	50	*P. verrucosum*	[286]
Blackberry	12h exposition to the compound in a paper towel	0.5, 1, 2, 5, and 10	Natural fungal growth.	[287]
Bread	Filter disk in Petri dishes with bread slices (10d of storage)	1 μL	*Rhizopus* *stolonifer*	[288]
Chicken breast	Multilayer carrageenan/chitosan coatings containing AITC, applied by immersion. Storage during 21d	20 and 200.	Natural fungal growth	[289]
Corn	Hydroxyethyl-cellulose gel disk	50	*Aspergillus* *flavus*	[285]
Grape	Injection of gaseous phase AITC on the first day (14d of total storage)	25 μg/mL	*A. niger*	[290]
Grape	Injection of gaseous phase AITC in the first day of storage (14d of total storage).	25 μg/mL	*A. carbonarius*	[290]
Grape	Injection of gaseous phase AITC in the first day of storage (14d of total storage).	25 μg/mL	*A. ochraceus*	[290]
Maize	Paper filter containing AITC during storage (30d)	0.125, 0.25, 0.5, 1, and 5	*A. flavus*	[291]
Maize	Paper filter containing AITC during storage (30d).	30 and 300.	*A. parasiticus*	[292]
Maize	Paper filter containing AITC during storage (30d)	30 and 300	*Fusarium* *verticillioides*	[292]
Maize	Paper filter containing AITC during storage (30d)	30 and 300	*F. graminearum*	[292]
Maize	Paper filter containing AITC during storage (15d).	25 μg/mL	*A. niger*	[290]
Maize	Paper filter containing AITC during storage (15d).	25 μg/mL.	*A. carbonarius*	[290]
Maize	Paper filter containing AITC during storage (15d).	25 μg/mL	*A. ochraceus*	[290]
Pita bread	Active packaging system containing AITC during storage (7d)	8, 16, 33 or 50 mg	*P. verrucosum*	[268]
Strawberry	Modified polyvinyl formal (PVFM) vibration-damping material	5%	*Botrytis cinerea*	[293]
Wheat	Hydroxyethyl-cellulose gel disk	50	*P. verrucosum*	[285]

## Data Availability

No new data were created or analyzed in this study. Data sharing does not apply to this article.
